# Subgroup analyses on return to work in sick-listed employees with low back pain in a randomised trial comparing brief and multidisciplinary intervention

**DOI:** 10.1186/1471-2474-12-112

**Published:** 2011-05-25

**Authors:** Christina M Stapelfeldt, David H Christiansen, Ole K Jensen, Claus V Nielsen, Karin D Petersen, Chris Jensen

**Affiliations:** 1 Department of Clinical Social Medicine, Centre of Public Health, Central Denmark Region and Department of Clinical Social Medicine and Rehabilitation, School of Public Health, University of Aarhus, Denmark; 2 Spine Centre, Department of Internal Medicine, Region Hospital Silkeborg, Denmark

## Abstract

**Background:**

Multidisciplinary intervention is recommended for rehabilitation of employees sick-listed for 4-12 weeks due to low back pain (LBP). However, comparison of a brief and a multidisciplinary intervention in a randomised comparative trial of sick-listed employees showed similar return to work (RTW) rates in the two groups. The aim of the present study was to identify subgroups, primarily defined by work-related baseline factors that would benefit more from the multidisciplinary intervention than from the brief intervention.

**Methods:**

A total of 351 employees sick-listed for 3-16 weeks due to LBP were recruited from their general practitioners. They received a brief or a multidisciplinary intervention. Both interventions comprised clinical examination and advice by a rehabilitation doctor and a physiotherapist. The multidisciplinary intervention also comprised assignment of a case manager, who made a rehabilitation plan in collaboration with the patient and a multidisciplinary team. Using data from a national database, we defined RTW as no sickness compensation benefit disbursement for four consecutive weeks within the first year after the intervention. At the first interview in the clinic, it was ensured that sick leave was primarily due to low back problems.Questionnaires were used to obtain data on health, disability, demographic and workplace-related factors. Cox hazard regression analyses were used with RTW as outcome measure and hazard rate ratios (HRR = HR_multidisciplinary_/HR_brief_) were adjusted for demographic and health-related variables. An interaction term consisting of a baseline variable*intervention group was added to the multivariable regression model to analyse whether the effects of the interventions were moderated by the baseline factor. Subsequently, a new study was performed that included 120 patients who followed the same protocol. This group was analyzed in the same way to verify the findings from the original study group.

**Results:**

The multidisciplinary intervention group ensured a quicker RTW than the brief intervention group in a subgroup with low job satisfaction, notably when claimants were excluded. The opposite effect was seen in the subgroup with high job satisfaction. When claimants were excluded, the effect was also in favour of the multidisciplinary intervention in subgroups characterised by no influence on work planning and groups at risk of losing their job. Inversely, the effect was in favour of the brief intervention in the subgroups who were able to influence the planning of their work and who had no risk of losing their job due to current sick leave. Interaction analysis of the data in the new study displayed similar or even more pronounced differences between subgroups in relation to intervention type.

**Conclusions:**

Multidisciplinary intervention seemed more effective than brief intervention in subgroups of patients with low job satisfaction, no influence on work planning and feeling at risk of losing their jobs due to their sick leave as compared with subgroups not fulfilling these criteria.

## Background

In Denmark, the costs of sick leave due to low back pain (LBP) reached approximately 3 billion Euro in 2007 which is equivalent to 22,500 employees being sick-listed full time for one year [1]. In a review of reviews, the following risk factors were consistently associated with slow return to work (RTW): functional disability, sciatica, older age, poor general health, psychosocial strain, negative cognitive characteristics, heavy physical work and receiving welfare payments [2]. However, the natural history of LBP is benign and self-limited in the majority of cases. The prognosis is therefore good and most sick-listed employees will return to work within six weeks [3,4]. The target population for rehabilitation are those who have not returned to work within a few weeks [3]. Long-term sick leave due to LBP is often rotted in a multitude of causes and multidisciplinary intervention is therefore recommended for rehabilitation of employees who are sick-listed for 4-12 weeks [5,6]. The efficacy of such interventions has not been consistently documented. Yet, intervention that involves a gradual RTW and workplace involvement has been shown to increase RTW rates in different settings and countries [7-10].

Some studies have argued for the identification of specific sub-groups of sick-listed employees who should be offered specific interventions. In a randomised controlled trial, sub-group analysis indicated that a work place intervention had more effect on older or high-risk employees than on younger people or employees without previous sick leave [11]. In another study, employees who had been sick-listed for at least eight weeks due to musculoskeletal pain were given scores assessing their chance of RTW [12]. Afterwards, they were randomly allocated to three different groups: a light multidisciplinary intervention group, an extensive multidisciplinary intervention group or a control group. Employees with a good prognosis showed similar RTW rates in the three groups, whereas employees with a poor prognosis returned to work significantly earlier when offered the extensive multidisciplinary treatment. Thus, it is important to study which kind of intervention is effective for specific subgroups of sick-listed employees, also referred to as one of the "Holy Grail"-type of questions by the Cochrane Back Review Group [13]. Successful RTW depends on factors related to the individual such as health, age, personality and family relations, but also on factors at work such as the psychosocial work environment and the interplay between individual and work-related factors. Multidisciplinary interventions typically rely on tailor-made "treatments" to facilitate RTW, which implies that job modifications or other RTW facilitation measures are only initiated if they are necessary. They often include efforts directed towards changing job demands, job control, work organisation or towards increasing support. This requires close collaboration between workplace stakeholders, the sick-listed employee, members of the multidisciplinary team and - in the Danish context - also the social service centre responsible for sick leave reimbursement. However, if the employee and the workplace stakeholders agree on job modifications or other arrangements without the involvement of external stakeholders, the multidisciplinary intervention teams will not be involved. It is therefore relevant to study if self-reported work-related factors may be used to predict if the RTW process would benefit from intervention by professionals in the fields of occupational and social factors.

In a recent randomised study, we compared the effects of a multidisciplinary intervention (with a focus on occupational and social factors) with those of a brief intervention (only health professionals involved) aimed at facilitating RTW for sick-listed employees with LBP [14]. After 12 months the two groups showed similar RTW rates, similar levels of reductions in disability, pain and fear avoidance scores, and the same improvement in general health scores. However, it may have been possible that specific sub-groups could have returned to work even earlier if they had received a particular kind of intervention. The objective of the present explorative study was to study whether particular subgroups identified on the basis of work-related factors would benefit more from the multidisciplinary than from the brief intervention.

After the original project had ended, sick-listed employees were included into a new study for another 12 months. We used the same intervention and randomisation procedures. A similar subgroup analysis was performed in the new study in order to test whether it was possible to reproduce the results from the original study group.

We hypothesized that particular subgroups defined by work-related factors would return earlier to work by a multidisciplinary than by a brief intervention if these work-related factors could be expected to influence the RTW process.

## Methods

### Study design and participants

The present paper was based on secondary analyses of prognostic factors in a randomised trial comparing a multidisciplinary and a brief intervention at 12-month follow-up [14]. The inclusion criteria were sick leave for 3-16 weeks due to low-back problems, 16-60 years of age and ability to read and speak Danish. Exclusion criteria were 1) unemployment; 2) continuing or progressive symptoms of spinal nerve-root affection implicating plans for surgery; 3) surgery in the spine within the past 12 months; 4) diagnosis of specific back disease (e.g. tumour); 5) diagnosis of primary psychiatric disease; 6) pregnancy; or 7) known substance abuse.

Patients from nine municipalities in Central Denmark Region were referred by their general practitioner (GP) based on the inclusion and exclusion criteria. The criteria were re-evaluated at The Spine Centre, Region Hospital Silkeborg, where the study was performed. All patients referred to the Spine Centre who adhered to the inclusion and exclusion criteria were included in the study. The total population in these nine municipalities was approximately 280,000 citizens.

After recruitment for the original study had closed, a second 12-month study was conducted with identical procedures and interventions. The original project was carried out from November 2004 to July 2007 and the inclusion of patients for the second study period started in August 2007 and ended in July 2008 (hereafter referred to as the new study).

### Interventions

Prior to randomisation, the participants completed a baseline questionnaire and underwent a thorough clinical examination by a rehabilitation doctor and a physiotherapist. The methods used for clinical examination have been described previously [14]. Reassuring explanations for pain and advice on a gradual increase in physical activity were provided. Subsequent randomisation was performed by a secretary on the basis of block randomisation generated by an externally located computer. At the following consultations, both participants and caregivers were aware of the result of the randomisation. Data analyses were performed by researchers outside the hospital. After two weeks, all participants were scheduled for a follow-up visit at the physiotherapist and usually also a follow-up visit at the rehabilitation doctor to inform the participant about the results of the magnetic resonance imaging (MRI) and other tests. After the first consultation, copies of the medical records were sent to the social service centre that was responsible for reimbursement of sick leave compensation All participants were free to contact the centre within the first three months.

For participants allocated to the brief intervention, care management stopped at the last visit at the physiotherapist or doctor. Treatment and rehabilitation were continued by the GP.

For participants allocated to the multidisciplinary intervention, a visit with the case manager was scheduled a couple of days after the first consultation. After a comprehensive interview covering aspects of work life and private life, a tailored rehabilitation plan was designed to facilitate the employee's RTW. The rehabilitation plan was discussed by the entire team at The Spine Centre. The team included a specialist of social medicine, a specialist of rehabilitation, a physiotherapist, a social worker and an occupational therapist. The case manager also contacted the work place and the social service centre to discuss and coordinate relevant initiatives. The case manager could arrange meetings between the participant and each of the other specialists, meetings at the work place and meetings with the social service centre, if relevant. A more complete description of the interventions is provided in a recent paper [14].

### Baseline variables

Work-related and basic socio-demographic variables were selected to cover well-known prognostic factors [2,15-20]. For the purpose of the present paper, baseline variables covered three domains: **1) Socio-demographics **including gender, age, marital status, parental status and education (*none, brief courses, skilled or trained, less than 3 years of education, bachelor degree, master degree*). **2) Work-related factors **including occupation in the public sector, work pace, support from colleagues and superior, job satisfaction, influence on work planning, shift work and interest in returning to the same job. **3) Combined health and work-related factors **including feeling at risk of losing job because of current sick leave, worrying about losing job because of medical conditions, whether LBP was work-related, work ability, permanent impairment of work ability, claim of compensation due to health problems, expectations of being back at work in six months *(numeric rating scale, 0-10)*, self-reported expectations of work ability in a year or desire to obtain incapacity benefit.

Finally, the questionnaire contained items on health and health-related factors, among others the SF36 instrument and the LBP rating scale [21]. The LBP rating Scale yields a score calculated as the sum of answers to six questions on worst, average and actual pain during the preceding two weeks for back and leg pain assessed on VAS box scales from 0 to 10 (sum score: 0-60).

### Outcome

The outcome measure was RTW, which occurred when the participant had not received sick leave compensation for a period of four consecutive weeks. Data on sick leave compensation were obtained from a national database administered by the Ministry of Employment. This database includes information on all public transfer payments for all Danish citizens registered on a weekly basis since 1991 [22]. Reasons for sick leave or other health data are not available in the database, but such information was obtained at the first interview in the clinic. At this meeting, it was confirmed that sick leave was due primarily to low back problems.

### Analyses

Subgroup analyses were based on tests for interaction. Statistical tests for interaction, which directly examine the difference in treatment effects between sub-groups, have been reported as a useful approach when performing subgroup analyses [23].

First, non-dichotomous baseline variables were dichotomised to have a sufficiently large number of participants in each group. Dichotomisation was data-driven for some of the variables and sensitivity analyses were carried out afterwards to check if associations were depending on the cut-off point.

Second, associations between dichotomised baseline variables and RTW were analysed and adjusted for age, gender and intervention. Hazard rate ratios (HRR) and 95% confidence intervals (95% CI) were calculated by using Cox regression analyses. The proportional-hazards assumption was assessed graphically using log-log plots adjusted for all covariates. The criterion was not fulfilled for the variables: "support from colleagues" and "claim of compensation due to health problems" wherefore these variables were not used in the further analyses.

A possible moderating effect of each baseline variable on the effect of intervention was identified in the next step by adding the interaction term "baseline factor*intervention" to a regression model with the baseline factor and intervention group adjusted for age and gender [23]. If the interaction term was significant with a p-value < 0.2, the baseline variable was included in the multivariable regression models to adjust for more baseline factors in the following step. Multivariable regression models were calculated within each of the domains: 1) socio-demographics, 2) work-related and 3) health and work-related factors. The interaction term was added in separate models for each baseline variable and the p-value was calculated for the interaction term. In this step, p < 0.05 was considered statistically significant. Besides the other baseline factors in the same domain, the interactions were adjusted for gender, age and health by including physical and mental component scores of SF36. However, in the combined health and work-related domain, the models were not adjusted for subjective health status to avoid over-adjustment as the baseline variables in this domain were directly related to health status. Cases with missing values in any of the baseline variables in each model were excluded from the analysis. The number of cases included in the analysis of each model is shown together with the HRRs (95% CI), which were calculated for the effect of multidisciplinary versus brief intervention for each subgroup.

Sick-listed employees who have claimed economic compensation for their disease or injury (claimants) have been shown to return later to work or show less symptom relief than employees who have claimed no compensation [24,25]. All sick-listed employees in Denmark are entitled to receive sickness compensation during their sick leave within the first year. Sometimes, it is possible to receive additional compensation if the health problem is caused directly by factors at work, such as heavy lifting. These cases were administered elsewhere, and RTW would often be unlikely before the issue of additional economic compensation was decided. In the present study, patients with an additional claim of compensation are called claimants. The effect of the interventions could differ between non-claimants and claimants within subgroups and this could dilute or strengthen interaction effects between interventions and work-related factors. In the present study, the subgroup analyses were therefore repeated in analyses without claimants. Claim status was reported by the participants in the baseline questionnaire.

In the analyses with all participants, 258 patients returned to work which left enough power for 17 parameters to be tested for interaction. None of the models contained more than 10 parameters. After excluding claimants, 173 subjects returned to work which indicated that we would have enough statistical power if we used a maximum of 11 parameters.

The same baseline factors as those used in the original project were tested in regression models with interaction terms based on data from the new study if the subgroups consisted of more than 20 patients. Claimants were not excluded in the analyses of the new study due to the low statistical power. Also, the lower number of subjects reduced the maximum number of parameters in the regression models. These models were therefore only adjusted for gender.

The software package, STATA 11.1, was used for statistical analyses.

### Ethical approval

The study was discussed with the regional research ethics committee. Approval was not considered necessary by the committee because all participants received the best available clinical care and because no biological material was involved. The study was performed in accordance with the Helsinki Declaration. All participants signed informed consent. The study was approved by the Danish Data Protection Agency (No. 2007-41-1278). The Trial Registration Number is ISRCTN18609003

## Results

### Participants

Baseline characteristics and the flow of the patients in the original project have been described previously [14]. In short, a total of 417 patients were referred to the study and 351 patients were included and randomised to brief (n = 175) or multidisciplinary intervention (n = 176). In the brief intervention group, 88 (50.3%) were women. In the multidisciplinary intervention group, 95 (54.0%) were women. In the two groups, the mean age was 41.9 (SD = 10.4) years and 42.1 (SD = 10.5) years, respectively. Seven participants did not continue after randomisation because of metastatic malignancy of the spine (n = 2), age (n = 1) or withdrawal (n = 4). This left 344 participants who completed the protocol. In the multidisciplinary intervention group, the case manager met the participants four times on average. Contacts with work place representatives were made in 87 cases (six times on average for each participant). The median duration of the intervention was 18 weeks in the multidisciplinary intervention group. In the brief intervention group, the participants were seen twice by the physiotherapist and once or twice by the rehabilitation doctor; a few participants were seen a few times more when needed.

Non-specific LBP was found in 191 (54%) patients. Radiculopathy was found in 112 participants (32%) and the remaining 48 participants (14%) were classified with other diagnoses (e.g. disc herniation without radiculopathy, spondylolisthesis). Later, 33 participants (9%) underwent surgery because of lack of improvement by conservative therapy (16 participants in the brief intervention group and 17 participants in the multidisciplinary intervention group). Mean pain levels on the LBP rating scale were 32.7 (SD = 12.4) in the brief and 31.6 (SD12.1) in the multidisciplinary intervention group.

### Predictors of RTW

During the first year of follow-up, 258 employees (74%) had returned to work. The remaining 93 subjects (26%) were still registered as receiving sickness benefits or other social transfer payments

(Table 1). None of the socio-demographic baseline variables were significantly associated with RTW (Table 2). Statistically significant associations with RTW were found for two of the work-related variables (Table 2). RTW was positively associated with willingness of colleagues to listen to their problems and influence on work planning. In the combined health and work-related domain, four variables were statistically significantly associated with RTW.

**Table 1:** The original and the new study groups.

	RTW				no RTW			
	Original study		New study		Original study		New study	
	%	n	%	n	%	n	%	n
**Socio-demographics**:								
Gender (% men)	49	258	42	83	44	93	41	37
Age (years, mean(SD))	41.4 (10.7)	258	40.5 (10.0)	83	43.6 (9.6)	93	41.3 (10.0)	37
Marital status (% single)	23	253	18	83	28	92	15	37
Children (% yes)	76	253	71	83	85	93	81	37
Education (%):		251		78		93		37
None	17		17		17		19	
Brief courses	12		9		16		16	
Skilled/trained	36		28		32		38	
< 3 years	12		13		13		5	
3-4 years	15		26		12		16	
> 4 years	3		4		2		3	
Other	5		4		8		3	
								
**Work-related factors**:								
Public employee (% yes)	41	246	44	78	43	89	27	37
High work pace (%):		253		83		93		37
Often	47		48		55		65	
Some times	47		43		37		30	
Seldom	5		8		8		3	
Never/hardly ever	1		0		1		3	
Support from colleagues (%):		250		81		93		37
Often	28		32		14		14	
Some times	52		52		56		49	
Seldom	16		12		19		22	
Never/hardly ever	4		4		11		16	
Colleagues willing to listen to work-related problems (%):		250		80		93		36
Often	49		53		38		44	
Some times	40		36		48		36	
Seldom	9		10		9		11	
Never/hardly ever	2		1		5		8	
Support from superior (%):		247		81		92		36
Often	29		38		28		22	
Some times	43		36		41		47	
Seldom	21		21		22		17	
Never/hardly ever	7		5		9		14	
Superior willing to listen to work-related problems (%):		246		81		92		37
Often	45		49		41		41	
Some times	38		31		30		30	
Seldom	11		17		21		13	
Never/hardly ever	6		2		8		16	
Job satisfaction, everything taken into consideration (%):		249		83		91		35
Very satisfied	46		47		40		29	
More or less satisfied	47		45		44		49	
Rather dissatisfied	6		7		12		20	
Very dissatisfied	1		1		4		3	
Influence on work planning (% yes)	80	251	79	82	67	90	43	35
Shift work (% yes)	21	251	27	82	29	90	25	36
Interested in returning to current job (% yes)	88	249	91	79	84	86	83	35
								
**Health and work-related factors**:								
Feeling at risk of losing job because of current sick leave (% yes)	35	246	27	81	49	86	34	35
Concerned about losing job because of medical condition (% yes)	52	252	41	82	60	90	46	35
This incidence of LBP is caused by my work (% yes)	55	250	60	83	61	90	75	36
Work ability, all in all (%)		251		80		92		35
Excellent	9		11		9		3	
Good	25		35		14		17	
Fairly good	27		18		26		34	
Fairly bad	27		22		23		23	
Very bad	12		14		28		23	
Permanently impaired work ability (% yes)	61	233	51		77	88	82	34
Claim of compensation due to health problems has been forwarded (% yes)	21	258	21	83	33	93	38	37
Chances of being back at work in 6 months (scale 0-10, mean(SD))	8.0 (2.6)	227	8.5 (2.0)	83	6.5 (3.1)	87	6.5 (2.9)	35
Work ability in a year (%):		250		82		89		35
Much better	34		43		28		14	
Fairly better	37		32		30		43	
More or less the same	26		25		34		40	
Fairly worse	3		0		8		3	
Much worse	1		0		0		0	
Incapacity benefit is desirable (%):		234		79		86		31
Absolutely not	91		96		77		77	
Maybe	8		4		16		16	
Absolutely	1		0		7		7	

Baseline characteristics of participants who returned to work (RTW) during the first 12 months and participants still sick-listed (no RTW) with low back pain after 12 months.

**Table 2:** Baseline predictors of RTW in original study group.

	Association with RTW	Interaction	Interaction
		All participants	Claimants excluded
	HRR (95% CI)*	P-value**	P-value**
**Socio-demographics**:			
Gender (men/women)	1.19 (0.93-1.52)	0.97	0.90
Age (<42/>42 years)	1.15 (0.90-1.97)	0.53	0.67
Marital status (married/single)	1.01 (0.75-1.36)	**0.17**	0.24
Children (no/yes)	1.25 (0.92-1.69)	0.23	0.21
Education (higher/lower)	1.05 (0.80-1.38)	0.35	0.56
			
**Work-related factors**:			
Public employee (yes/no)	1.06 (0.80-1.41)	0.92	0.72
High work pace (not often/often)	1.21 (0.94-1.54)	0.71	0.82
Colleagues willing to listen to work-related			
problems (often/not often)	**1.33 (1.03-1.72)**	0.27	0.63
Support from superior (not often/often)	1.04 (0.79-1.37)	0.41	0.77
Superiors willing to listen to work-related			
problems (often/not often)	1.14 (0.88-1.47)	0.48	0.63
Job satisfaction (very/not very satisfied)	1.18 (0.91-1.51)	**0.020**	**0.026**
Influence on work planning (yes/no)	**1.40 (1.03-1.90)**	**0.16**	**0.14**
Shift work (no/yes)	1.24 (0.91-1.68)	0.64	0.99
Interested in returning to current job (yes/no)	1.22 (0.83-1.81)	**0.13**	**0.13**
			
**Health and work-related factors**:			
Risk of losing job because of current			
sick leave (no/yes)	1.30 (1.00-1.69)	0.27	**0.040**
Concerned about losing job because of medical condition (no/yes)	1.19 (0.93-1.53)	0.33	**0.14**
LBP is caused by my work (no/yes)	1.05 (0.81-1.35)	0.28	0.39
Work ability, all in all (good/bad)	1.24 (0.96-1.60)	0.39	0.27
Permanently impaired work ability (no/yes)	**1.67 (1.28-2.18)**	**0.048**	0.27
Back at work in 6 months? (sure/not sure)	**1.62 (1.24-2.11)**	0.84	0.43
Work ability in a year (better/not better)	**1.38 (1.05-1.82)**	0.68	0.53
Incapacity benefit is desirable (no/yes)	**2.04 (1.29-3.23)**	0.55	0.33

*adjusted for gender, age and intervention

**adjusted for gender and age

HRR (hazard rate ratio) is shown for each baseline variable without analysis of interaction in first column. In second column, P-values are shown for the interaction between baseline variable and intervention. P-values for the interaction between baseline variables and intervention are also shown after exclusion of 83 participants who have claimed compensation. All variables were dichotomised and analysed with Cox regression.

### Subgroups

In the first step, using all subjects in the analyses of univariables, we found significant interactions adjusted for age and gender (p < 0.20) between the type of intervention and marital status, job satisfaction, influence on work planning, interest in returning to the same job and feeling permanently impaired regarding work ability (Table 2). After exclusion of the 83 claimants, statistically significant interaction was seen for risk of losing job and concerned about losing job. In the next step in which multivariable models were adjusted for age, gender and other baseline factors, the subgroup with high job satisfaction in the brief intervention group returned earlier to work than the corresponding subgroup in the multidisciplinary intervention; and the effect was the opposite in the subgroup with low job satisfaction, especially when claimants were excluded (Table 3). When claimants were excluded, the effect was also in favour of the multidisciplinary intervention in subgroups characterised by no influence on work planning and at risk of losing their job, whereas the effect was in favour of the brief intervention for the subgroups who had influence on work planning and no risk of losing job due to current sick leave (Table 3).

**Table 3:** Effect of multidisciplinary team-intervention compared with brief intervention in subgroups from the original study group

	All participants			Claimants excluded		
	HRR (95% CI)	P of interaction	n	HRR (95% CI)	P of interaction	n
Socio-demographic models**						
No moderator*	0.88 (0.68-1.13)			0.93 (0.70-1.23)		
Married	0.79 (0.59-1.05)	0.28	250	0.84 (0.61-1.17)	0.40	193
Single	0.95 (0.55-1.64)		83	1.02 (0.56-1.87)		64
						
Work-related models**						
No moderator*	0.85 (0.66-1.11)			0.95 (0.70-1.28)		
Job satisfaction, high	**0.52 (0.35-0.76)**	**0.002**	142	**0.44 (0.27-0.71)**	**0.001**	106
Job satisfaction, low	**1.25 (0.88-1.78)**		175	**1.49 (1.00-2.23)**		137
						
Influence on work planning	0.76 (0.57-1.03)	0.06	243	**0.83 (0.59-1.16)**	**0.036**	187
No influence on work planning	1.23 (0.67-2.25)		74	**1.33 (0.68-2.62)**		56
						
Interested in returning to current job	0.76 (0.57-1.01)	0.10	275	0.82 (0.59-1.13)	0.052	206
Not interested in returning to current job	1.45 (0.65-3.25)		42	1.63 (0.68-3.89)		37
						
Health and work-related models***						
No moderator*	0.90 (0.69-1.17)			1.03 (0.76-1.39)		
At risk of losing job because of current sick leave	1.15 (0.73-1.83)	0.17	117	**1.60 (0.94-2.75)**	**0.031**	88
Not at risk of losing job	0.77 (0.56-1.08)		190	**0.78 (0.54-1.15)**		146
						
Worried about losing job	1.02 (0.70-1.47)	0.28	165	1.25 (0.81-1.90)	0.19	126
Not worried about losing job	0.76 (0.51-1.11)		142	0.79 (0.51-1.25)		108
						
Permanently impaired work ability	0.97 (0.69-1.37)	0.47	200	1.13 (0.76-1.69)	0.38	148
Not permanently injured	0.76 (0.48-1.20)		107	0.89 (0.54-1.47)		86

*HRR was calculated in the multivariable model without the interaction term

**Adjusted for gender, age and physical and mental component scores (SF36).

***Adjusted for gender and age.

HRR (HR_multidisciplinary group_/HR_brief intervention_) was calculated for each level of the independent variable.

In the above-mentioned analyses, job satisfaction was dichotomised into the categories "very satisfied" and "very dissatisfied to more or less satisfied". Alternatively, if "more or less satisfied" and "very satisfied" had defined the best category, the worst category, "very to rather dissatisfied" would have shown an even stronger difference in effect of the two interventions (HRR = 3.26 (95% CI: 1.03-10.3, n = 30). Interaction effects of age were not present, either when the cut-point for dichotomisation was moved to a lower or when it was moved to a higher age. Other sensitivity analyses showed no different patterns of interaction effects for any of the baseline variables.

### Combined subgroups

**Figure 1:**
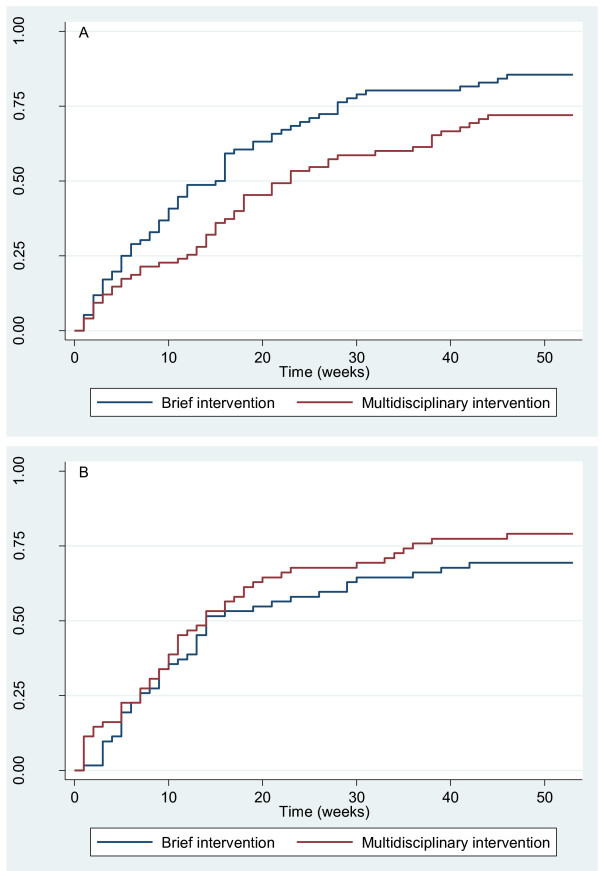
**Return to work (RTW) of subgroups in the original study group**. Participants with compensation claims were excluded. Fraction of participants with RTW is shown during follow-up. The first visit at the clinic is at week 0. A. Subgroup of 144 participants with influence on work planning and no risk of losing their job. B. Subgroup of 117 participants without influence on work planning and/or at risk of losing their job.

The baseline variables that demonstrated interaction in relation to the interventions were correlated. Thus, a relatively high fraction of those who were not satisfied with their job or had no influence on work planning felt at risk of losing their job. For instance, among those without influence on work planning, 69% felt that they were at risk of losing their job. Among those who had influence on work planning, only 29% felt that they were at risk of losing their job. In both cases, participants with compensation claims were not included. A subgroup comprising participants with influence on work planning and not feeling at risk of losing their job returned to work earlier if they had received the brief intervention (HRR = 0.65 (95% CI: 0.45-0.95), n = 144, Figure 1A) than if they had received the multidisciplinary intervention. The effect of the interventions was reversed in the subgroup comprising participants without influence on work planning and/or at risk of losing their job (HRR = 1.42 (95% CI: 0.92-2.18), n = 117, Figure 1B) as the interaction term was statistically significant (p = 0.008).

### New study

In the new study, 120 patients were included (60 patients in each group). Women accounted for 58.3% of the participants, men for 41.7%. The women's mean age was 41.5 (SD = 10.1), the men's 39.9 (SD = 9.8) years. The mean pain level on the LBP rating scale was 35.2 (SD = 11.7) in the brief and 35.1 (SD12.1) in the multidisciplinary intervention group.

The HRR of multidisciplinary versus brief intervention in the new study was 1.14 (95% CI: 0.74-1.76).

The subgroup analyses presented in Table 3 were repeated in the new study except for those groups that were stratified with respect to marital status and interest in returning to the same job, since one strata of each of these two variables consisted of less than 20 subjects. Thus, five variables remained for subgroup analyses in the new study (Table 4). For the two work-related factors, the HHRs of multidisciplinary versus brief intervention were similar to those obtained in the original study, i.e. favourable effects of the multidisciplinary intervention in subgroups with low job satisfaction and no influence on job planning were supported by similar hazard ratios in the new study. Those at risk of losing their job due to current sick leave, those worrying about losing their job and those with a permanently impaired work ability showed higher HHRs of multidisciplinary versus brief intervention in the new study group, but the interaction was not statistically significant.

**Table 4:** Effect of multidisciplinary team-intervention compared with brief intervention in the new study group.

	HRR (95% CI)	P of interaction	n
			
No moderator*	1.14 (0.74-1.76)		120
			
Work-related models**			
Job satisfaction, high	0.72 (0.38-1.38)	0.14	49
Job satisfaction, low	1.41 (0.77-2.57)		69
			
Influence on work planning	0.95 (0.58-1.54)	0.48	80
No influence on work planning	1.49 (0.57-3.93)		37
			
Health and work-related models**			
At risk of losing job because of current sick leave	1.95 (0.78-4.88)	0.10	34
Not at risk of losing job because of current sick leave	0.95 (0.57-1.59)		82
			
Worried about losing job	1.84 (0.93-3.64)	0.11	50
Not worried about losing job	0.87 (0.49-1.54)		67
			
Permanently impaired work ability	1.40 (0.82-2.38)	0.47	71
Not permanently injured	0.93 (0.41-2.11)		42

*HRR was calculated in the multivariable model without the interaction term

**Adjusted for gender.

HRR (HR_multidisciplinary group_/HR_brief intervention_) was calculated for each level of the independent variable.

**Figure 2:**
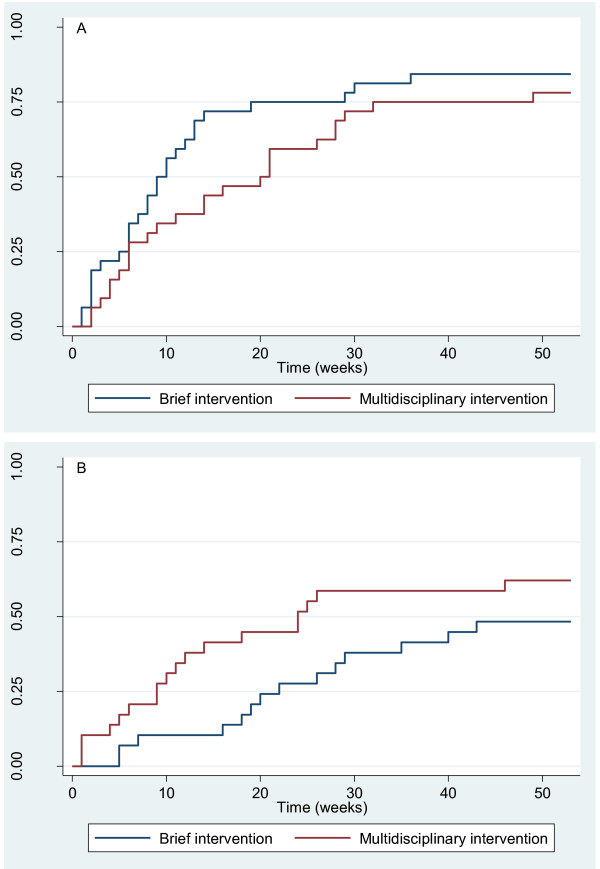
**Return to work (RTW) of subgroups in the new study group**. Fraction of participants with RTW is shown during follow-up. The first visit at the clinic is at week 0. A. Subgroup of 62 participants with influence on work planning and no risk of losing their job. B. Subgroup of 56 participants without influence on work planning and/or at risk of losing their job.

The combined subgroup in the new study of those with influence on work planning and no risk of losing their job also tended to return earlier to work if they received the brief intervention (HRR = 0.73 (95% CI: 0.41-1.28), n = 62, Figure 2A). In the new study, the other combined subgroup comprising participants without influence on work planning and/or at risk of losing their job returned to work earlier if they received the multidisciplinary intervention (HRR = 2.16 (95% CI: 1.03-4.53), n = 56, Figure 2B). The interaction term in the combined subgroup analysis was statistically significant (p = 0.025).

## Discussion

A number of predictors of RTW were found, but only one variable, "job satisfaction", significantly modified the effects of the interventions in the multivariable model in which all participants were included. When claimants were excluded, statistically significant interaction effects were found for another two variables: "influence on work planning" and "feeling at risk of losing one's job due to the present sick leave". Thus, participants with low job satisfaction, no influence on work, no interest in returning to the same job and at risk of losing their job seemed to return earlier to work when they received the multidisciplinary intervention, whereas participants without these characteristics returned to work earlier when they received the brief intervention.

The new independent study also showed no difference in RTW between the two interventions, and, furthermore, it supported most of the effects found in the subgroups of the original project. Only 120 patients participated in the new study and, our focus was therefore more on the estimates of the hazard ratios than on the statistical tests. The interaction effects were also found in the new study for the variables "job satisfaction", "influence on work planning" and "feeling at risk of losing one's job due to the present sick leave". In the last case, the hazard ratio for the subgroup "feeling at risk" was considerably higher, which indicates an even greater benefit of the multidisciplinary intervention than in the original study for this subgroup. Also for the variable "worried about losing one's job", a considerably stronger interaction estimate was found in the new study than in the original study. Thus, the quality of the interventions may have changed; or changes may have occurred on the labour market. The new study was carried out at the beginning of the global financial crisis at which time more patients were worried about losing their jobs than during the original project period which was characterized by an economic boom.

The modifying effects of subgroups on intervention effects may be explained in different ways: It may have occurred either by statistical chance, or it may have reflected true causal relationships. The latter is considered more probable as the associations were confirmed in the new study. However, it should also be considered whether these associations were, indeed, plausible; that is, we must ask ourselves whether the interventions could be expected to show different effects in these subgroups. Experts in occupational and social factors were only involved in the multidisciplinary intervention, whereas health professionals were involved in both kinds of intervention and provided care and treatment for all participants according to the hospital's clinical standards. It would therefore be expedient to search for facilitation of RTW by the multidisciplinary intervention in subgroups needing assistance to perform their job, to make arrangements with their employer or other occupational issues. For instance, "those feeling at risk of losing their jobs" may have benefitted more from the collaboration between occupational or social professionals and the employer than those not at risk. Employees not at risk may feel more confident about their health and their ability to come to an agreement with their employer regarding job modification. A similar explanation may exist for the interacting effect between influence on work planning and the type of intervention. Influence on work planning may facilitate low back patients' RTW, for example if the patients are allowed to take breaks when needed, to change work tasks and to reduce heavy lifting or if they are allowed influence on other types of job modification [2,19,20,26]. For employees with low job control, this may have been achieved better through intervention by an occupational therapist of the multidisciplinary team than by the brief intervention where this was not possible. Modifying effects were also observed for job satisfaction. However, an explanation for this is less straightforward given that we have no information about the reasons for job dissatisfaction. Nevertheless, the correlation between the baseline variables indicated that low job control could contribute to low job satisfaction, which was not surprising. We also expected to see other subgroups, such as those with low social support from a supervisor or those with high work pace, benefit more from a multidisciplinary than from a brief intervention, but this was not the case. The backdrop against which the present study of a wide range of work-related factors was launched was the lack of previous studies on very specific work-related issues that should be considered by multidisciplinary teams. Some of these factors apparently affected the outcome of the multidisciplinary effort, whereas others were not important. This should be further examined in new studies.

An ongoing compensation claim is a risk factor for not returning to work [19,25]. In the present study, the associations between baseline characteristics and RTW differed for those with and without an ongoing compensation claim. Consequently, statistically significant interaction effects were not fully equivalent for the same variables before and after excluding claimants. Strong evidence is needed to obtain additional compensation and it may be necessary to document that LBP was directly caused by physical work loads. It may take several months or more than a year to come to a decision on this type of compensation. In some cases, sick-listed employees do not RTW before such a decision is made. This may delay RTW for all participants with a claim. The clearer identification of effects in subgroups without claimants than in subgroups where all participants were analysed indicated that the delay was greater in the multidisciplinary than in the brief intervention group. Thus, the HRR seemed to increase in subgroups benefitting from the multidisciplinary intervention when claimants were excluded.

Subgroups that modify the effect of biopsychosocial interventions have been analysed in a few studies. In a Norwegian study [12], a screening instrument was deployed to test whether patients sick-listed for at least eight weeks because of musculoskeletal pain would benefit more from a light or from an extensive multidisciplinary intervention. Those most likely benefitting from the extensive intervention were characterized by poor prognosis, such as having more complaints if work was continued, limited control on their work situation and difficulty turning down tasks at work or at home. The extensive multidisciplinary intervention included occasional workplace interventions and associations were reported similar to those found in the present paper. A light multidisciplinary intervention seemed sufficient for patients with medium or good prognosis. This intervention was similar to the brief intervention used in our study and the interventions studied elsewhere [27,28]. Low-intensive back school has also been reported to have the same or a slightly better effect on return to work than high-intensive back school [29]. The moderating effects of beliefs about reduced ability to work on the effects of intervention and control groups were observed in a study by Hagen et al. [30] at three months follow-up. However, their intervention did not include a work place intervention and the moderating effect was not present after one year [30].

A moderating effect of age has previously been reported. However, using almost the same cut-off point as in the studies by Steenstra et al. [11] and Hagen et al. [30], we were unable to reproduce their results. The lack of a control group in our study, i.e. both groups received an intervention, may explain the lack of a moderating effect of age. The other modifying variables identified in the studies by Steenstra and Hagen, (e.g. sick leave in previous year [11], gastrointestinal complaints at baseline [30]) were not analysed in our study.

The dichotomisation was data-driven for some of the baseline variables. This may increase the risk of reporting spurious results or associations that occurred by chance. However, most participants used the two best categories of the baseline variables to describe their situation which left little choice for other cut-points than between these to categories. Also, the sensitivity analyses showed no patterns that would contradict the findings reported here. In fact, in the case of job satisfaction, a "lower" cut-point revealed an even stronger effect of the multidisciplinary intervention. This indicated that the different effects of the multidisciplinary and the brief interventions may be most pronounced for employees with the most negative or positive ratings of work-related factors. Another potential problem is that of multi-co-linearity between work-related factors. It is clear that it was often the same participants who assessed that they had low social support, low job control, low work ability and so forth. However, these factors do measure different aspect of working life and we were interested in identifying as many factors as possible with a modifying effect. The correlation between independent variables increased the risk of confounding, which was minimised by adjusting for other baseline factors in the multivariable regression models. The statistical power was relatively low for analyses of interaction. We therefore decided to adjust only for baseline factors within the same domain, i.e. the work-related or the health and work-related domain. This procedure should have eliminated the most likely candidates for confounding, but could not rule out that other factors confounded the results. A drawback of the attempt to identify as many factors as possible was the large number of analyses and the associated risk of reporting spurious results. Thus, 5% of all associations would be expected to be statistically significant by chance. This risk was acknowledged by the re-analysis and confirmation of results in a new sample of sick-listed employees, which indicated that the different effects in subgroups did not occur by chance.

However, the statistical power was low and the interaction effects were not statistically significant in the new study. Furthermore, the HRRs within specific subgroups were not consistently different from 1, even when the interaction was statistically significant. The test for interaction only revealed differences between the HRRs of two mutually exclusive subgroups. The subgroups composed of the combined subgroups of 1) no influence on work planning/risk of losing job and 2) influence on work planning/no risk of losing job was an example of this. Both in the original study and in the new study, the interaction of these subgroups on the intervention effect was statistically significant. However, in the original study, the HRR was significantly lower than 1 (brief intervention more effective) in the subgroup with influence on work planning/no risk of losing job, whereas the HRR was not significantly higher than 1 in the other subgroup. In the new study, the HRR was significantly higher than 1 (multidisciplinary intervention more effective) in the subgroup with no influence on work planning/at risk of losing job, whereas the HRR was not significantly lower than 1 in the other subgroup.

RTW was based on data from a national register considered to be valid [31] and in which 100% follow-up is ensured. However, the register has limitations. The data on social transfer payments were estimated on a weekly basis, e.g. less than a week's payment will be displayed as a whole week's payment. This may result in an overestimation of the time until RTW. As the follow-up period was one year, it was considered a minor problem; and, moreover, one that would affect the two intervention groups equally.

Thus, the risk of reporting erroneous results due to mass significance, problems with multi-co-linearity and bias in post-hoc analyses was reduced by conducting similar analyses with similar results in a new study. However, the next and final step would be to conduct a new randomised trial with specific hypotheses and advance stratification by relevant subgroups.

## Conclusion

Multidisciplinary intervention seemed more effective than brief intervention in subgroups of patients with low job satisfaction, no influence on work planning and feeling at risk of losing their jobs due to their sick leave as compared with subgroups with high job satisfaction, influence on work planning and no perceived risk of losing their jobs. The findings were confirmed in a new subset of patients receiving similar interventions.

## Competing interests

The authors declare that they have no competing interests.

## Authors' contributions

CMS and CJ conceived the study, carried out the statistical analyses and drafted the manuscript.

OKJ and DHC were involved in providing the interventions.

All authors participated in the design of the study, helped draft the manuscript, helped improve the analyses and interpret the results. All authors have approved the final manuscript.

## Pre-publication history

The pre-publication history for this paper can be accessed here:

http://www.biomedcentral.com/1471-2474/12/112/prepub

## References

[B1] Kjøller M Juel K Kamper-Jørgensen F The Public Health Report Denmark 2007 2007 Copenhagen, The National Institute of Public Health (SIF), University of Southern Denmark 1 482

[B2] Hayden JA Chou R Hogg-Johnson S Bombardier C Systematic reviews of low back pain prognosis had variable methods and results: guidance for future prognosis reviews J Clin Epidemiol 2009 62 781 796 10.1016/j.jclinepi.2008.09.004 19136234

[B3] Waddell G The back pain revolution 2004 2 Elsevier Limited

[B4] Zampolini M Bernardinello M Tesio L RTW in back conditions Disabil Rehabil 2007 29 1377 1385 10.1080/09638280701314980 17729083

[B5] Karjalainen K Malmivaara A van TM Roine R Jauhiainen M Hurri H Multidisciplinary biopsychosocial rehabilitation for subacute low back pain in working-age adults: a systematic review within the framework of the Cochrane Collaboration Back Review Group Spine (Phila Pa 1976) 2001 26 262 269 10.1097/00007632-200102010-00011 11224862

[B6] Waddell G Burton AK Concepts of rehabilitation for the management of low back pain Best Pract Res Clin Rheumatol 2005 19 655 670 10.1016/j.berh.2005.03.008 15949782

[B7] Anema JR Steenstra IA Bongers PM de Vet HC Knol DL Loisel P Multidisciplinary rehabilitation for subacute low back pain: graded activity or workplace intervention or both? A randomized controlled trial Spine (Phila Pa 1976) 2007 32 291 298 10.1097/01.brs.0000253604.90039.ad 17268258

[B8] Bultmann U Sherson D Olsen J Hansen CL Lund T Kilsgaard J Coordinated and tailored work rehabilitation: a randomized controlled trial with economic evaluation undertaken with workers on sick leave due to musculoskeletal disorders J Occup Rehabil 2009 19 81 93 10.1007/s10926-009-9162-7 19169654

[B9] Loisel P Abenhaim L Durand P Esdaile JM Suissa S Gosselin L A population-based, randomized clinical trial on back pain management Spine (Phila Pa 1976) 1997 22 2911 2918 10.1097/00007632-199712150-00014 9431627

[B10] Norlund A Ropponen A Alexanderson K Multidisciplinary interventions: review of studies of return to work after rehabilitation for low back pain J Rehabil Med 2009 41 115 121 10.2340/16501977-0297 19229442

[B11] Steenstra IA Knol DL Bongers PM Anema JR van MW de Vet HC What works best for whom? An exploratory, subgroup analysis in a randomized, controlled trial on the effectiveness of a workplace intervention in low back pain patients on return to work Spine 2009 34 1243 1249 10.1097/BRS.0b013e3181a09631 19412140

[B12] Haldorsen EM Grasdal AL Skouen JS Risa AE Kronholm K Ursin H Is there a right treatment for a particular patient group? Comparison of ordinary treatment, light multidisciplinary treatment, and extensive multidisciplinary treatment for long-term sick-listed employees with musculoskeletal pain Pain 2002 95 49 63 10.1016/S0304-3959(01)00374-8 11790467

[B13] Bouter LM Pennick V Bombardier C Cochrane back review group Spine (Phila Pa 1976) 2003 28 1215 1218 10.1097/01.BRS.0000065493.26069.1C 12811262

[B14] Jensen C Jensen OK Christiansen DH Nielsen CV One-year follow-up study of a controlled clinical trial using light mobilisation and an informative approach to low back pain: Randomised clinical trial comparing multidisciplinary and brief intervention Spine in press

[B15] Crook J Milner R Schultz IZ Stringer B Determinants of occupational disability following a low back injury: a critical review of the literature J Occup Rehabil 2002 12 277 295 10.1023/A:1020278708861 12389479

[B16] Fayad F Lefevre-Colau MM Poiraudeau S Fermanian J Rannou F Wlodyka DS [Chronicity, recurrence, and return to work in low back pain: common prognostic factors] Ann Readapt Med Phys 2004 47 179 189 15130717 10.1016/j.annrmp.2004.01.005

[B17] McIntosh G Frank J Hogg-Johnson S Hall H Bombardier C Low Back Pain Prognosis: Structured Review of the Literature Journal of Occupational Rehabilitation 2000 10 101 115 10.1023/A:1009450102876

[B18] Shaw WS Pransky G Fitzgerald TE Early prognosis for low back disability: intervention strategies for health care providers Disabil Rehabil 2001 23 815 828 10.1080/09638280110066280 11763278

[B19] Steenstra IA Verbeek JH Heymans MW Bongers PM Prognostic factors for duration of sick leave in patients sick listed with acute low back pain: a systematic review of the literature Occup Environ Med 2005 62 851 860 10.1136/oem.2004.015842 16299094 PMC1740930

[B20] Turner JA Franklin G Turk DC Predictors of chronic disability in injured workers: a systematic literature synthesis Am J Ind Med 2000 38 707 722 10.1002/1097-0274(200012)38:6<707::AID-AJIM10>3.0.CO;2-9 11071692

[B21] Manniche C Asmussen K Lauritsen B Vinterberg H Kreiner S Jordan A Low Back Pain Rating scale: validation of a tool for assessment of low back pain Pain 1994 57 317 326 10.1016/0304-3959(94)90007-8 7936710

[B22] Hedegaard J DREAM database. [14] 2007 The National Labour Market Authority

[B23] Assmann SF Pocock SJ Enos LE Kasten LE Subgroup analysis and other (mis)uses of baseline data in clinical trials Lancet 2000 355 1064 1069 10.1016/S0140-6736(00)02039-0 10744093

[B24] Atlas SJ Chang Y Kammann E Keller RB Deyo RA Singer DE Long-term disability and return to work among patients who have a herniated lumbar disc: the effect of disability compensation J Bone Joint Surg Am 2000 82 4 15 10653079 10.2106/00004623-200001000-00002

[B25] Rasmussen C Leboeuf-Yde C Hestbaek L Manniche C Poor prognosis in back pain among patients who have filed financial claims - secondary publication Ugeskr Laeger 2009 171 1604 1607 19425255

[B26] Johansson G Lundberg I Adjustment latitude and attendance requirements as determinants of sickness absence or attendance. Empirical tests of the illness flexibility model Soc Sci Med 2004 58 1857 1868 10.1016/S0277-9536(03)00407-6 15020004

[B27] Indahl A Velund L Reikeraas O Good prognosis for low back pain when left untampered. A randomized clinical trial Spine (Phila Pa 1976) 1995 20 473 477 10.1097/00007632-199502001-00011 7747232

[B28] Molde HE Grasdal A Eriksen HR Does early intervention with a light mobilization program reduce long-term sick leave for low back pain: a 3-year follow-up study Spine (Phila Pa 1976) 2003 28 2309 2315 10.1097/01.BRS.0000085817.33211.3F 14560075

[B29] Heymans MW de Vet HC Bongers PM Knol DL Koes BW van MW The effectiveness of high-intensity versus low-intensity back schools in an occupational setting: a pragmatic randomized controlled trial Spine (Phila Pa 1976) 2006 31 1075 1082 10.1097/01.brs.0000216443.46783.4d 16648740

[B30] Hagen EM Svensen E Eriksen HR Predictors and modifiers of treatment effect influencing sick leave in subacute low back pain patients Spine (Phila Pa 1976) 2005 30 2717 2723 10.1097/01.brs.0000190394.05359.c7 16371893

[B31] Hjollund NH Larsen FB Andersen JH Register-based follow-up of social benefits and other transfer payments: accuracy and degree of completeness in a Danish interdepartmental administrative database compared with a population-based survey Scand J Public Health 2007 35 497 502 10.1080/14034940701271882 17852980

